# A Randomised, Double-Blind, Controlled Efficacy Trial of the LiESP/QA-21 Vaccine in Naïve Dogs Exposed to Two *Leishmania infantum* Transmission Seasons

**DOI:** 10.1371/journal.pntd.0003213

**Published:** 2014-10-09

**Authors:** Gaetano Oliva, Javier Nieto, Valentina Foglia Manzillo, Silvia Cappiello, Eleonora Fiorentino, Trentina Di Muccio, Aldo Scalone, Javier Moreno, Carmen Chicharro, Eugenia Carrillo, Therese Butaud, Laurie Guegand, Virginie Martin, Anne-Marie Cuisinier, David McGahie, Sylvie Gueguen, Carmen Cañavate, Luigi Gradoni

**Affiliations:** 1 Department of Veterinary Clinical Sciences, University Federico II, Naples, Italy; 2 Leishmaniasis and Chagas Disease Unit, Centro Nacional de Microbiología, Instituto de Salud Carlos III, Majadahonda, Spain; 3 Unit of Vector-borne Diseases and International Health, MIPI Department, Istituto Superiore di Sanità, Rome, Italy; 4 Safety and Efficacy Unit, Biological R&D, VIRBAC BIO3, Carros, France; 5 Medical Department, VIRBAC, Carros, France; National Institutes of Health, United States of America

## Abstract

Canine leishmaniasis is an important zoonosis caused by uncontrolled infection with *Leishmania infantum*, where an inappropriate immune response is not only responsible for permitting this intracellular parasite to multiply, but is also responsible for several of the pathological processes seen in this disease. Effective canine vaccines are therefore a highly desirable prevention tool. In this randomised, double-blinded, controlled trial, the efficacy of the LiESP/QA-21 vaccine (CaniLeish, Virbac, France) was assessed by exposing 90 naïve dogs to natural *L. infantum* infection during 2 consecutive transmission seasons, in two highly endemic areas of the Mediterranean basin. Regular PCR, culture, serological and clinical examinations were performed, and the infection/disease status of the dogs was classified at each examination. The vaccine was well-tolerated, and provided a significant reduction in the risk of progressing to uncontrolled active infection (p = 0.025) or symptomatic disease (p = 0.046), with an efficacy of 68.4% and a protection rate of 92.7%. The probability of becoming PCR positive was similar between groups, but the probability of returning to a PCR negative condition was higher in the vaccinated group (p = 0.04). In conclusion, we confirmed the interest of using this vaccine as part of a comprehensive control program for canine leishmaniasis, and validated the use of a protocol based on regular in-depth assessments over time to assess the efficacy of a canine leishmaniasis vaccine.

## Author Summary

Canine leishmaniasis is caused by uncontrolled infection with *Leishmania infantum*. The dog is also the principle reservoir of this parasite which can infect humans. The key determinant of the outcome of infection is the ability of the dog's immune system to respond appropriately. This should be mainly cell-mediated immunity; the antibody responses often desired for viral or bacterial infections are not protective. Therefore, vaccines capable of inducing this cell-mediated immune profile are a desirable prevention tool. Previous studies with CaniLeish (consisting of the parasite's excreted-secreted proteins with an adjuvant) have demonstrated the ability of this vaccine to induce an appropriate immune profile and to protect against an intravenous challenge, as well as to reduce the risk of transmission of the parasite to the sandfly vector. This paper reports a study where vaccinated dogs were exposed to natural parasite transmission over two full seasons in two Mediterranean locations (one in Italy and one in Spain). Data from the unvaccinated control group confirmed that the levels of exposure were high. We demonstrated that the vaccine significantly reduces the risk of progressive infections and disease confirming the interest of using vaccination as part of a comprehensive control program for this disease.

## Introduction

Canine leishmaniosis (CanL) is an extremely variable and polymorphic disease, caused by progressive uncontrolled infection with the intracellular parasite *Leishmania infantum* (in the Old World, synonym of *L. chagasi* in the New World) transmitted by the bites of phlebotomine sand flies [1]. It is endemic in the Mediterranean basin, Middle East, Central Asia and Latin America [2] where the dog is considered to be the main reservoir host for zoonotic infection [3]. In Europe, the infection is usually transmitted by members of the *Phlebotomus (Larroussius)* subgenus, although non-vectorial transmission is also rarely reported [4]–[6]. Several topical insecticide preparations are licensed in Europe and provide a useful reduction in the frequency of infection [7]. However as these cannot prevent all potentially infectious bites there is still a need for further control measures [8], [9], and recent data suggest that although they have good potential efficacy when correctly used, in the hands of owners they are not sufficiently effective [10].

The outcome of infection with *L. infantum* is unpredictable and some dogs will completely resolve the infection, while others will go on to develop CanL after a highly variable incubation period [9], [11]. In addition, dogs that do go on to develop the disease also have a wide range of clinical presentations, ranging from mild papular skin disease or only mild clinicopathological abnormalities through to severe generalised disease characterised by renal failure and death [12], [13]. Even when dogs appear to be controlling the parasite, the situation may not be stable long term, and immunosuppression or other intercurrent diseases may permit subpatent infections to become progressive months or even years later [1], [9]. For these reasons it is important to note that infection is not equal to disease for this parasite [9].

The determining factor of the outcome of infection is the ability of the immune system to manage the parasite efficiently. It is now generally accepted that resistance to developing CanL is primarily dependent on whether or not the dog develops an appropriate T-helper (Th)1-dominated cell-mediated immune response against the parasite [14], [15]. This appropriate immune response can reduce the parasitic burden in the macrophage by means of Nitric Oxide production after stimulation with Th1-type cytokines such as IFN-γ [16], [17] and thus prevent progression of the infection. Furthermore, much of the pathological impact of the disease is caused by the combination of an inappropriate, ineffective Th2-dominated response in the face of uncontrolled parasite growth [18].

The development of the disease therefore correlates with increasing parasite burdens and a strong but ineffective humoral response [9]. It also correlates with a reduced delayed-type hypersensitivity response to intradermal *Leishmania* antigen exposure which is a marker of a depressed cell-mediated immune response to the parasite [19] and to the level of infectivity of dogs to sand flies [20], [21]; symptomatic dogs being more infective to insect vectors than asymptomatic dogs [22], [23]. Furthermore, even after treatment, dogs can still harbour the parasite and remain infectious to sand flies [24], [25].

Therefore, when the immune system can correctly manage the infection this will reduce the risk of progressive infections that result in disease or the death of the animal and should also reduce the contribution of the dog to the ongoing spread of the parasite in the local area. For these reasons several authors have expressed the opinion that an effective vaccine against CanL could be the best control strategy for both canine and human disease [8], [26], and there is a growing consensus that an ideal control program for CanL is likely to involve combined use of vaccines with repellent products to maximize the protection of the dog [27], [28].

In recent years two CanL vaccines have been registered in Brazil. Both have a primary course consisting of three injections, followed by annual booster injections. Leishmune (Zoetis, Brazil) is based on the fucose-mannose ligand of *L. donovani* in association with a saponin adjuvant and demonstrated 76% efficacy against disease or death after natural infection in a field study with evidence of a type 1 immune response being provided by positive intradermal skin test results [29]. The other vaccine available in Brazil is LeishTec (Hertape Calier, Brazil) which uses the recombinant A2 antigen of *L. chagasi* in association with a saponin adjuvant, and demonstrated 43% protection against a culture positive state in an artificial challenge model [30]. However, until recently no vaccines were available in Europe.

With the launch of the LiESP/QA-21 vaccine (CaniLeish, Virbac, France), such an option has been made available in Europe for the first time. Previous studies have demonstrated that vaccination with LiESP/QA-21 induces an appropriate Th1-dominated cell-mediated immune response within three weeks of completing the primary course and that this response remains effective for a full year and is capable of reducing the parasite load in pre-infected macrophages *in vitro* [31], [32]. The immune profile induced by the vaccine is characterised by a significant increase in the specific cell-mediated immune response, and also the production of specific antibodies with a predominant IgG2 profile that are detected by conventional IFAT assays [32], [33]. It has also been demonstrated that LiESP/QA-21 vaccination reduces the parasite load and the risk of progressive infection in vaccinated dogs when they receive an intravenous promastigote challenge one year after completing the primary course [33]. However, during natural transmission there is also a potential impact of sand fly salivary proteins [34], and so experimental challenge studies are unable to definitively determine the efficacy of such a vaccine.

In this paper, we report results of a randomised, double-blinded, controlled 2-season natural-challenge trial with the LiESP/QA-21 vaccine performed in highly endemic areas of the Mediterranean basin. The objective of the study was to assess the ability of the vaccine to reduce the incidence of active infections, both asymptomatic and symptomatic, in the face of intense exposure to natural challenge.

## Materials and Methods

### Ethics Statement

All study procedures were approved by the National Authorities in Italy and Catalonia (Spain). The study design and technical protocol of investigations were approved by the Veterinary Board of the Italian Ministry of Health following the European Directive 86/609/EEC, adopted by the Italian Government with the Law 116/1992 and by the Department of Biodiversity and the Environment of the Government of Catalonia under number 6760 in accordance with Spanish law on the protection of animals used for experimentation and other scientific purposes (Royal Decree 1201/2005 and Law 32/2007). The Spanish legislation is a transposition of Directive 86/609/EEC.

### Study Area, Population and Design

Two study sites in rural areas with seasonal transmission were selected based on historical records demonstrating extremely high natural transmission levels: one in the Naples province in southern Italy and one near Barcelona in northern Spain. The activity period of the main local phlebotomine vector, *Phlebotomus perniciosus*, ranges between the end of May and early October. In the Naples area, *L.infantum* infection rates in dissected sand flies were found to range from 2.8–6.1% [35]. In cohorts of exposed naïve dogs, the incidence of *L.infantum* infection and CanL clinical disease was reported to average about 40% and 20%, respectively, after exposure to 2 transmission seasons [36]. In the Barcelona area, 0.5% of *P.perniciosus* females were found to be infected by *L.infantum* promastigotes, whereas seroprevalence in dogs was found to range from 2–10% in random sampling studies and 7–21% in kennels [37].

In the interest of the “three Rs” of animal welfare (Replace, Reduce, Refine), the number of dogs was kept to the minimum consistent with significance of expected results. Sample size calculation was performed assuming the following parameters: 80% infection rate; 40% culture positivity rate; 75% vaccine efficacy at P<0.05. Hence, 90 conventional, *Leishmania*- and *Ehrlichia*-naïve Beagle dogs (49 males and 41 females), aged 5 to 7.5 months were randomly split into two groups (46 vaccinees and 44 controls) according to sex, weight, age and litter. They had all previously received their routine conventional vaccinations (DHPPiLR) and deworming treatments. The dogs were born in non-endemic areas, and all were confirmed to be PCR negative (on bone marrow samples) and seronegative for *Leishmania* immediately before the vaccination phase commenced. The primary vaccination phase was performed under controlled and protected laboratory conditions (to avoid any potential contact with sand flies). Three to four weeks after the final dose of the primary vaccination course, the dogs were transferred to the study sites (late June for Barcelona and mid-July for Naples to ensure rapid exposure to challenge). The date of the transfer was designated Month 0 (M0). All dogs were once again confirmed to be PCR negative for *Leishmania* at M0. The groups were equally divided by sites to ensure the equivalent composition per group at each site (23 vaccinated and 22 control dogs per site, 24 males and 21 females for Barcelona and 25 males and 20 females for Naples). The dogs were kept in open kennels for 24 months. Regular deworming treatments were administered; the use of antiparasitic drugs or insecticides/repellents against sand flies was prohibited to allow maximum natural exposure of the animals to sandfly bites. The study followed the Good Clinical Practice guidelines. All analyses and clinical examinations were performed in a blinded manner by professionals who had access only to the dog identification codes. Treatment group codes were only unblinded after all data had been entered into a data-management system and all decisions regarding the status of each dog had been taken.

### Vaccine and Vaccination Protocol

The LiESP/QA-21 vaccine is commercially available in the European Union under the trade name CaniLeish (Virbac, France). It is composed of purified excreted-secreted proteins (ESP) of *Leishmania infantum* (LiESP) produced by means of a patented cell-free, serum-free culture system invented by the IRD (Institut de Recherche pour le Développement) [38] and adjuvanted with QA-21, a highly purified fraction of the *Quillaria saponaria* saponin. The doses used in this study were formulated as for the commercial product.

The dogs assigned to the vaccinated group received one dose of vaccine every 21 days for a total of three doses at the start of the study, and then an annual booster during the natural exposure period. Control dogs were not vaccinated.

### Vaccine Safety

Dogs were kept under regular veterinary surveillance during the entire study. The occurrence of potential adverse reactions to the vaccine was particularly assessed by daily clinical examinations for three days post-injection with assessment of local and general clinical signs (local pain, pruritus, swelling or nodules, fever etc.), followed by a general examination once a week during the initial vaccination phase. Any adverse reactions noted after annual booster injections were also recorded.

### Serology Testing of the Humoral Immune Response

ELISA testing was used during the vaccinal phase (on the day of each dose of the primary vaccination course and 2 weeks after the third dose) to dose the level of IgG1 and IgG2 antibodies to both ESP and also specifically to Parasite Surface Antigen (PSA), which is a major antigenic component of ESP. The technique was performed as previously described [31], [32] using serial three-fold dilutions of the serum to be tested, from 1/150 to 1/12150. Dogs were considered as negative when the titre was inferior to 1/450.

Immunofluorescence testing (IFAT) was also performed on the same samples to dose the level of total anti-*Leishmania* antibodies with the exception of the samples obtained at the second and third dose of the primary vaccination course. Briefly, *L. infantum* parasites are fixed to slides, then different dilutions of the serums to be tested are deposited on the slides. After 30 minutes of incubation at 35–37°C serum antibodies fixed to the parasites are revealed by immunofluorescence using a secondary fluorescent anti-IgG antibody. The titre corresponds to the last dilution for which at least 50% of the parasites display visible fluorescence. Dogs were considered as infected when the titre was ≥1/160. Titres in the range of 1/40–1/80 were considered to be indicative of *Leishmania* exposure in the control dogs [18].

### Clinical Follow-up during Challenge

Every three months during the challenge phase (M0 to M24), and on the day of the annual vaccine booster, the dogs were weighed and examined for symptoms attributable to CanL: deterioration of the general state, fever, weight loss, poor body condition, muscle atrophy (in particular temporal or occipital), digestive disorders, PU/PD, palor of mucosae, skin disorders (ulcers/nodules, furfur, onychogryphosis, erythema, pruritis, alopecia, hyperkeratosis), sensory disorders, lymphadenopathy, splenomegaly, ocular disorders (blepharitis, conjunctivitis, keratitis, uveitis) and arthritis.

### Paraclinical Follow-up during Challenge

Haematological and biochemical parameters (platelet, white and red blood cell counts, albumin/globulin ratio and total proteins) were also monitored every three months to detect typical alterations: leucopenia, anaemia and/or thrombocytopenia, hyperproteinemia (total protein>9 g/l) and/or hyperglobulinemia (inverted A/G ratio <0.9). The results were compared to standard values obtained from two reference texts [39], [40].

### Parasitological Follow-up during Challenge

Parasite detection was performed using both PCR and culture in blood-agar media on M0, M9, M15 and then every three months until M24.

PCR was performed on bone marrow samples as previously described, using both the small subunit ribosomal ribonucleic acid gene with a nested technique [11], [41], and also a 200 bp DNA fragment of the kinetoplast minicircles with a RT-PCR technique for which the results are expressed as parasites per ml bone marrow [33]. Contaminations of amplicons were avoided by using physical separation (rooms, materials) as well as decontamination procedures (UV exposure and bleaching of materials and surfaces). Bone-marrow samples from *Leishmania*-free dogs were used as negative controls at each step of the procedure.

Parasite isolation by culture was performed using NNN medium on bone marrow samples in Barcelona, and Evans' modified Tobie's medium (EMTM) on lymph node aspirates in Naples. The technique was as previously described [11], [41].

### Assessment of Challenge Level

The exposure rate was defined as the percentage of animals in the control group demonstrating infection with *L. infantum* by means of a positive result on PCR and/or culture, or showing any IFAT titre ≥1/40 during the course of the study.

### Dog Status Classification

**Figure 1:**
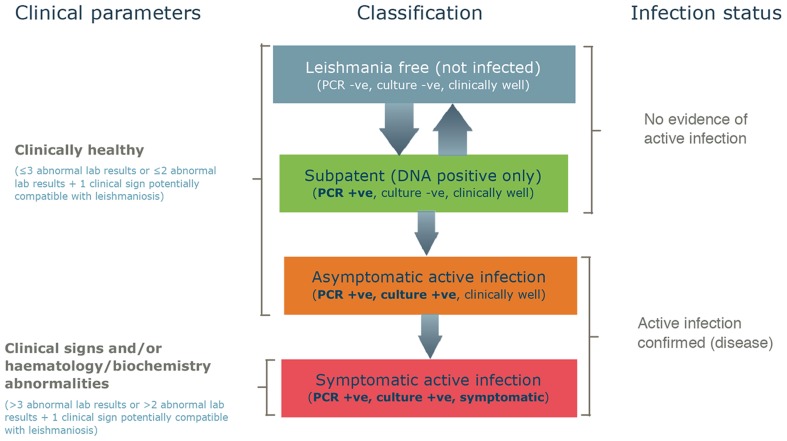
Classification of the infection status of the dogs. This system of classification was used at each full clinical assessment and at the end of the study to determine the status of each dog.

At each clinical assessment and at the completion of the study the classification of the dogs' *Leishmania* status was determined using the results of the parasitological tests, the other laboratory tests and the presence or absence of clinical signs as presented in figure 1. Subpatent infections were defined by the transient or sustained detection of parasite DNA in bone marrow from dogs with negative tissue culture results. Active infections were defined by the detection of parasite growth in tissue culture from PCR-positive dogs, shortly followed by the elevation of IFAT titers. Active infections were then further sub-classified as symptomatic (clinical score>3) or asymptomatic (clinical score ≤3). This classification system was adapted from various references in the literature [9], [18], [42]–[44].

The following criteria were considered for the calculation of the clinical score:

clinical score ≤3: a maximum of three abnormal parameters (laboratory abnormal data, such as altered full blood count, total proteins and albumin/globulin ratio, and/or clinical signs attributable to CanL).
clinical score>3: more than three 3 abnormal parameters (laboratory abnormal data, such as altered full blood count, total proteins and albumin/globulin ratio, and/or clinical signs attributable to CanL).

### Euthanasia Endpoint

According to the ethical requirements of the study protocol, euthanasia was performed for sick dogs that showed severe clinical signs such as emaciation, severe sensorial depression and dehydration due to renal involvement. All dogs euthanized were then submitted to necropsy.

### Statistical Analysis

Analyses were performed with the software SAS V9.1. The significance threshold was α = 0.05. The site effect was taken into account in the statistical tests.

The status of the vaccinated and control dogs was compared at M24 when the efficacy of the vaccine was calculated using the standard efficacy calculation: (%symptomatic controls - %symptomaticvaccinated)/% symptomatic controls * 100. The status of any dog which had died due to CanL was carried forward to this point. The percentage of non-symptomatic vaccinated animals was reported as the protection rate.

Frequencies and percentages of symptomatic dogs were compared between groups using a Cochran-Mantel-Haenszel test to examine the association between symptomatic animals and treatment group while adjusting for the site (or study) effect. The same analysis was performed for the dogs with an active infection (i.e. both symptomatic and asymptomatic dogs with positive culture). A categorical analysis was performed for the three categories: Symptomatic/Asymptomatic/Subpatent or Free. Frequencies and percentages of each category were compared between groups using a Cochran Armitage Trend test. The following scores were attributed to the different categories: 1 for the category “Symptomatic Active Infection”, 2 for the category “Asymptomatic Active Infection” and 3 for the categories “Subpatent” or “Free”. These scores were calculated for each group and compared using a non-parametric Wilcoxon test.

The survival curves of the vaccinated and control groups were compared using a log-rank test. The events were defined by the first time the dog became actively infected and the first time the dog became symptomatic for the two analyses respectively. Percentages of death per group were compared using a Fisher's exact test.

A comparison of the RT-PCR values obtained from M9 to M24 for both groups was performed by non parametrical repeated measures analysis with group, time, site, site×group interaction, site×time interaction and group×time interaction as fixed effects and dogs as a random effect. The Last Observation Carried Forward (LOCF) method was used for missing RT-PCR values.

## Results

### Vaccine-Related Adverse Events

The LiESP/QA-21 vaccine was well-tolerated. Benign local swelling, occasionally associated with pain, was the most remarkable adverse event observed after injection. It was observed in several dogs after the second and third injections (15 and 23 dogs respectively), and resolved spontaneously within 2 to 8 days. One dog presented crusting at the injection site after the first and the second injections. This resulted in transient focal alopecia. No nodules or ulcerations were recorded. Vaccination was not associated with fever, lymphadenopathy or any other general reaction.

### Trial Population for Analysis

Ten dogs (five per site, consisting of 3 control and 2 vaccinated dogs from one site and 2 control and 3 vaccinated dogs from the other site), died during the course of the natural challenge period from causes unrelated to CanL. As their leishmanian status could not be determined at the end of the study, they were excluded from the efficacy analysis and retained only for the safety analysis. Deaths were attributed to transfer between vaccination and challenge sites for one dog, acute endometritis for one dog, pulmonary oedema associated with septicaemia for one dog, fatal accident for one dog, hemorrhagic enteritis of unknown etiology for one dog and a chronic syndrome of infectious origin (possibly *Leptospira*) for four dogs (2 per group). In one case, the cause of the death remained undetermined.

As a result, 80 dogs (39 controls and 41 vaccinated) were retained for the efficacy analysis. Dogs that were euthanized or died due to CanL had their last observations carried forward for the purposes of efficacy analysis.

### Serological Responses to Vaccination

#### ELISA testing of anti-ESP and anti-PSA IgG1 and IgG2

**Figure 2:**
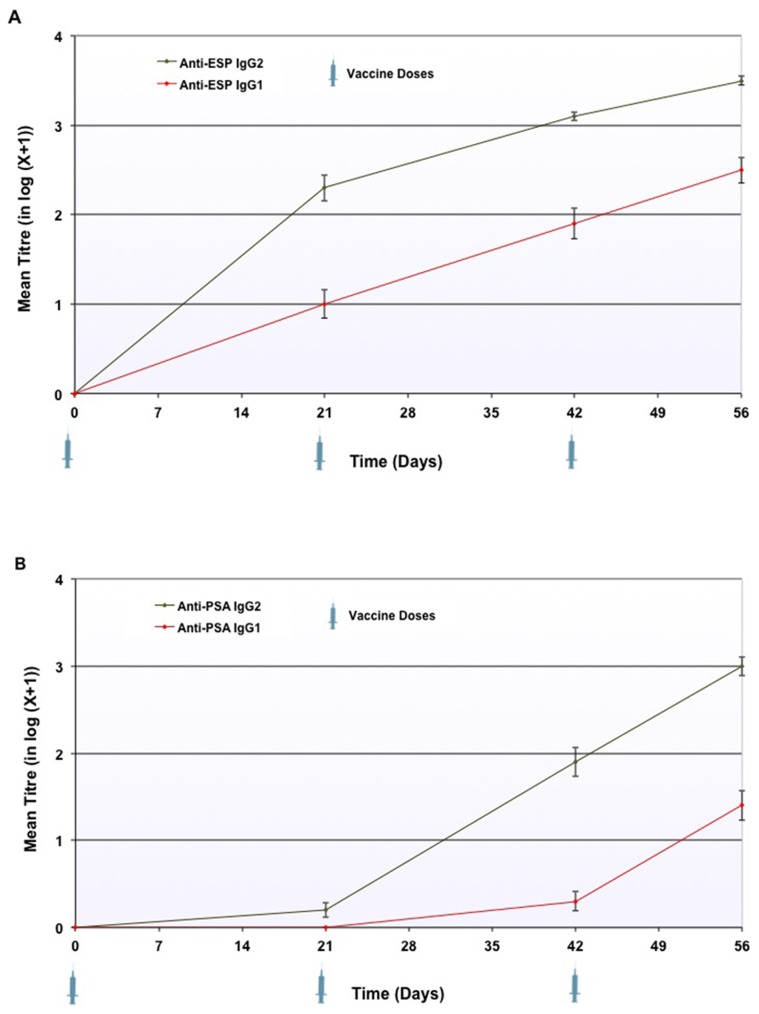
Progression in log-transformed anti-ESP IgG1 and IgG2 reciprocal titres (Panel A) and anti-PSA IgG1 and IgG2 reciprocal titres (Panel B) in vaccinated dogs during the vaccinal period. The technique was performed using serial three-fold dilutions of the serum to be tested, from 1/150 to 1/12150. Dogs were considered as negative when the titre was inferior to 1/450. Dogs with a negative result were regarded as having a result of zero after log conversion of the reciprocal titres to allow this to be represented on the charts. Error bars represent the standard error of the mean.

Over the course of the vaccinal phase, all LiESP/QA-21 immunised dogs developed an IgG2 response to ESP (range 1/450 to 1/12150). 96% of the vaccinated population (44/46) developed an IgG2 response to PSA (range 1/450 to 1/12150). The details are presented in Figure 2. By contrast, 89% of the vaccinated dogs (41/46) developed an IgG1 response to ESP (range 1/450 to 1/4050) and 57% (26/46) developed an IgG1 response to PSA (range 1/450 to 1/4050) by day 56. These details are also presented in Figure 2. The serological response to vaccination with LiESP/QA-21 was therefore characterised by an IgG2-dominated profile.

No antibodies against ESP or PSA were observed in the control group at any time point during the vaccinal period.

#### Total IgG response against L. infantum assessed by IFAT

70% of the vaccinated dogs (32/46) were at or above the diagnostic threshold for the IFAT assay (range 1/160 to 1/1280) by day 56. This confirms that antibodies induced by vaccination can lead to positive results when using conventional IFAT in vaccinated dogs.

### Exposure Rate

90% of the control dogs (95% in Naples (18/19) and 85% in Barcelona (17/20)) demonstrated *Leishmania* exposure (by IFAT titres of at least 1/40 or a PCR positive state) on at least 1 occasion during the course of the study confirming the high levels of transmission at the sites. Confirmed infection by culture and/or PCR, was demonstrated in 72% (28/39) of the control dogs.

### Incidence and Progression of Leishmanial Infections

**Figure 3:**
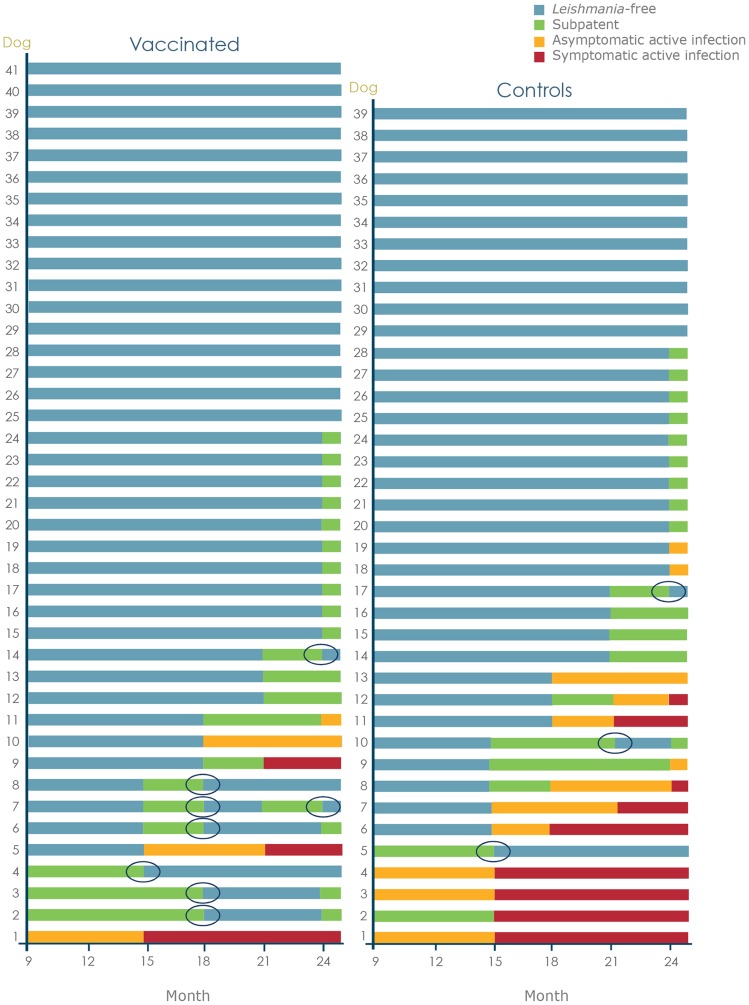
Longitudinal record of the infection status of each dog between months 9 and 24. The dogs were classified at each time point using a system based on PCR, culture and clinical signs and clinicopathological parameters (the details of this system are given in figure 1). Points at which subpatent dogs converted back to a *Leishmania*-free (PCR negative) status are circled.

The infection status of each dog over the course of the study is charted in figure 3. There was no significant difference between groups in the proportion of dogs becoming PCR positive on at least 1 occasion, confirming that the vaccine does not prevent initial entry and migration of the parasite. Dogs with the subpatent status that could be followed to a subsequent check point showed occasional conversion to the *Leishmania*-free status, this being more frequent in the vaccinated group (p = 0.0396). These events are circled in figure 3. None of the dogs that developed asymptomatic active infection showed spontaneous conversion to a subpatent condition. Similarly, none of the dogs with symptomatic infection showed spontaneous resolution of clinical signs, confirming the progressive nature of the disease.

**Figure 4:**
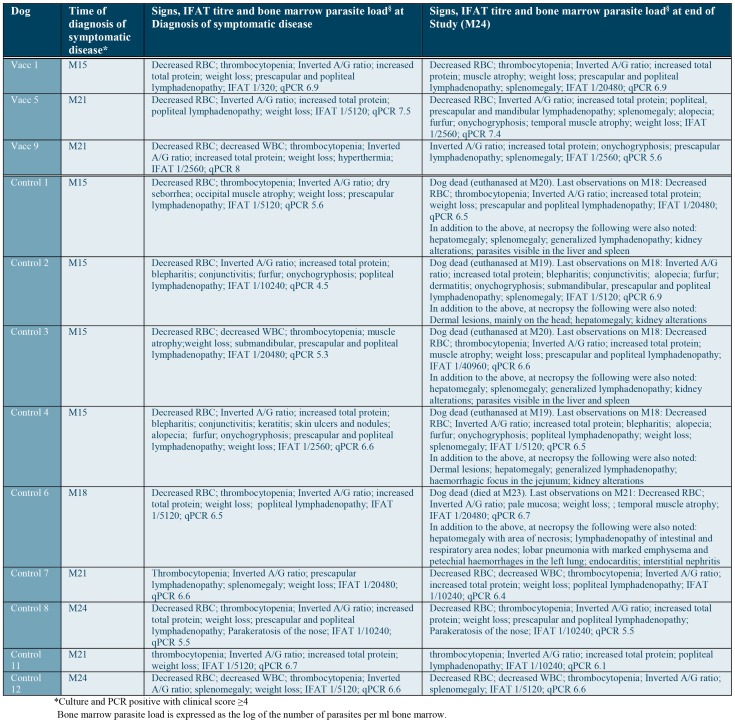
Clinical findings, laboratory abnormalities and IFAT titres seen in dogs diagnosed with symptomatic infections at the time of the diagnosis and at the end of the study.

There was a difference between groups in the development of the more severe, fatal stages. Five dogs in the control group either died or were euthanized due to CanL during the study. Severe CanL was confirmed on necropsy for all of these dogs. No dogs in the vaccinated group died or were euthanized due to CanL during the study, although one dog was euthanized some days after the M24 final assessment (p<0.0001). When only dogs reaching the symptomatic stage were considered to assess the speed of progression of symptomatic disease to fatal disease within the study duration this difference remained significant (p<0.0001). In addition, dogs in the vaccinated group reaching the symptomatic active infection status were primarily classified as symptomatic based on altered laboratory parameters such as blood cell counts, whereas in the control group the clinical signs were generally more obvious and severe as noted in Figure 4. This confirms that even when dogs in the vaccinated group progressed to the symptomatic disease it was generally slower and less severe.

**Figure 5:**
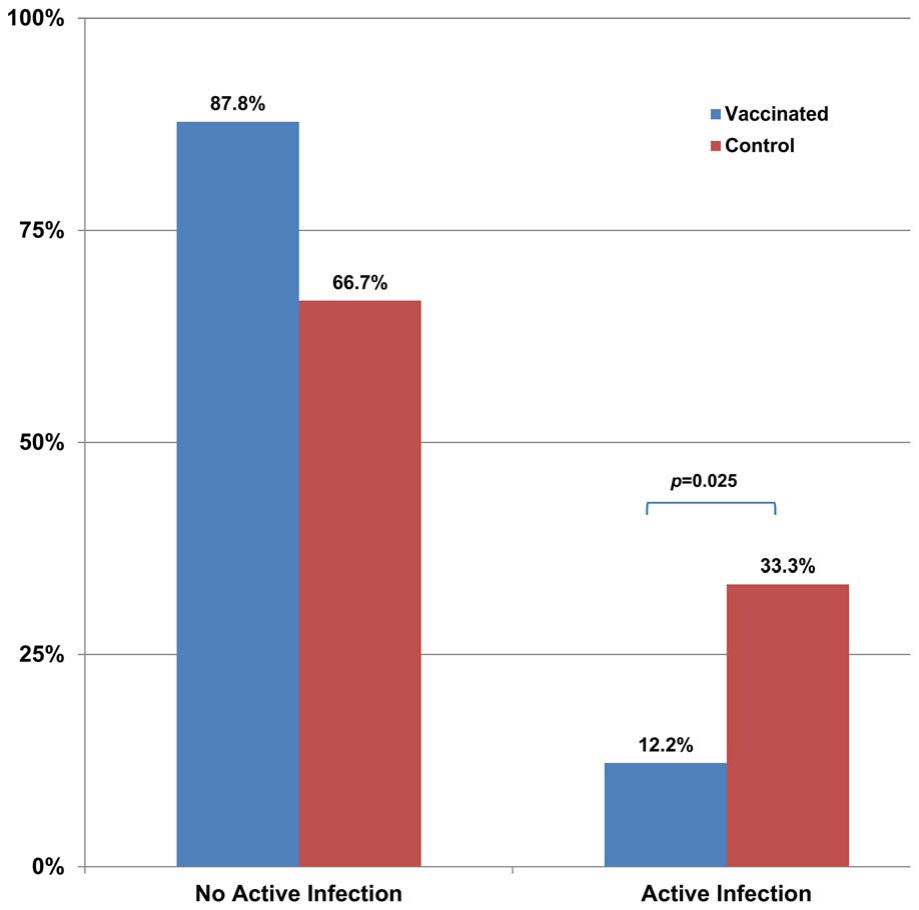
Comparison of the proportion of dogs in each group that progressed to active infection by the end of the 2 year study. Active infection was determined by positive results with PCR and biphasic medium culture with or without clinical signs.

**Figure 6:**
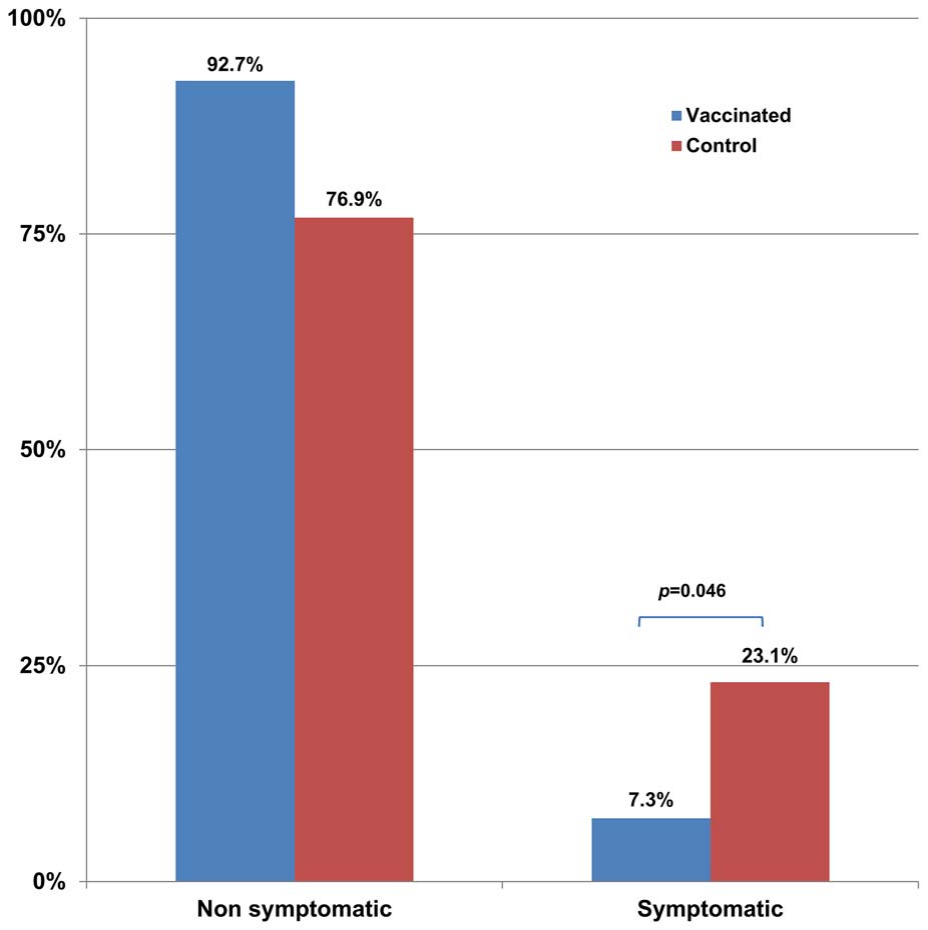
Comparison of the proportion of dogs in each group that progressed to symptomatic CanL by the end of the 2 year study. Symptomatic CanL was defined as active infection with the presence of a clinical score>3.

By M24, 33.3% of the dogs in the control group were demonstrating active infection; this was significantly different from the vaccinated group, with 12.2% (p = 0.025) as shown in figure 5. The number of symptomatic cases also differed significantly between the vaccinated (7.3%) and control (23.1%) groups (p = 0.046) as shown in figure 6.

The efficacy of the vaccine in the prevention of clinical signs of CanL in this study is therefore 68.4%, with a protection level of 92.7%.

When the prevention of clinical disease is expressed as an odds ratio, this equates to an odds ratio of 3.8 between the groups.

**Figure 7:**
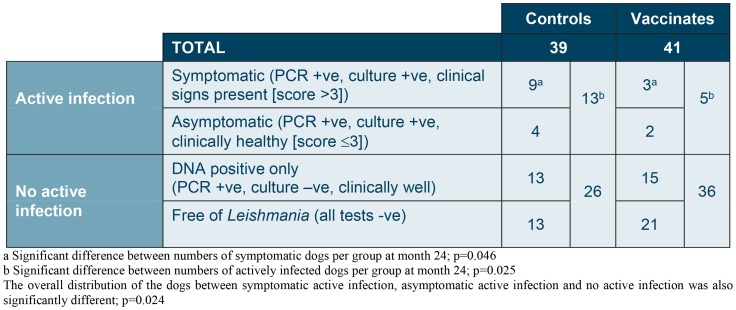
Summary of the dogs' Leishmania status at month 24 after two years of exposure in a highly endemic environment.

The distribution of dogs between all stages (symptomatic, asymptomatic, subpatent and *Leishmania*-free) was also significantly different between vaccinated and control groups (p = 0.024). The details are presented in Figure 7.

**Figure 8:**
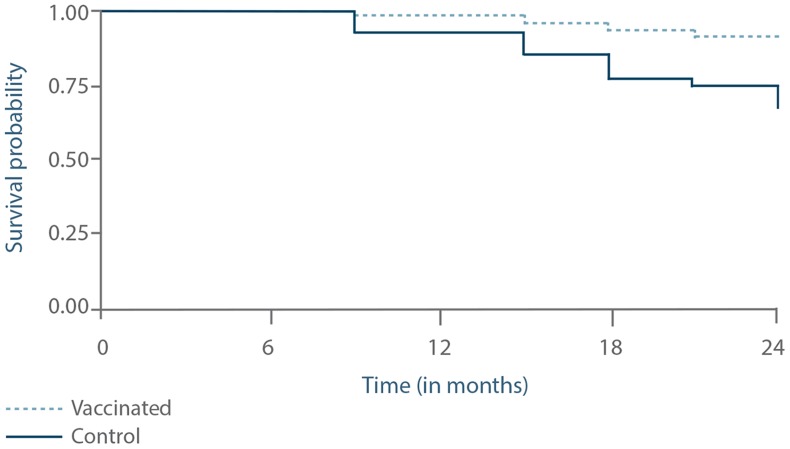
Progression of the proportion of dogs with active infection as assessed by a survival curve analysis. The status of the dogs was assessed at months 9, 15, 18, 21 and 24 using the classification system in figure 1. Active infection was identified by positive PCR *and* positive culture. The two groups were significantly different (p = 0.0265).

Survival curve analysis of the progression to active infections (asymptomatic or symptomatic) is presented in figure 8. This differed significantly between the two groups, with a higher probability to develop active infections in the control group than in the vaccinated group (p = 0.0265).

**Figure 9:**
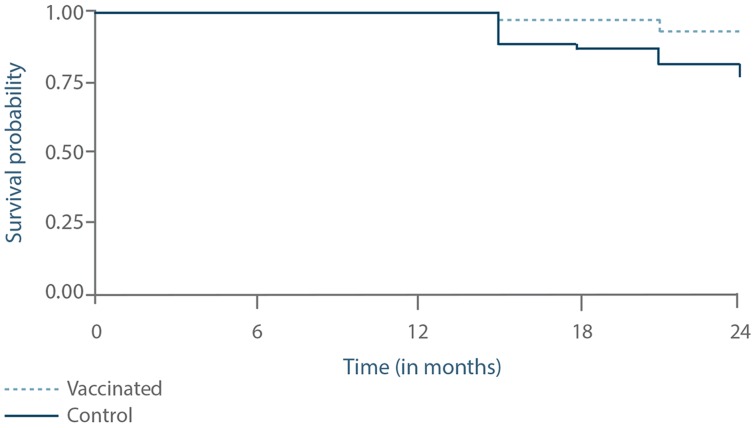
Progression of the proportion of dogs with symptomatic infection as assessed by a survival curve analysis. The status of the dogs was assessed at months 9, 15, 18, 21 and 24 using the classification system in figure 1. Symptomatic infection was identified by positive PCR and positive culture associated with a clinical score>3. The two groups were significantly different (p = 0.0466).

Likewise, as presented in figure 9, survival curve analysis for progression to symptomatic disease was significantly different between the control and vaccinated groups, with a higher probability to develop symptomatic CanL in the control group than in the vaccinated group (p = 0.0466).

**Figure 10:**
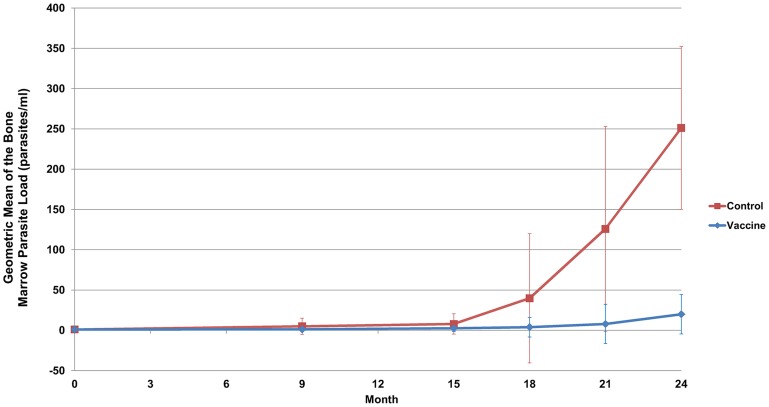
Geometric means of the bone marrow parasite loads over the course of the study. The bone marrow parasite loads were assessed by quantitative RT-PCR targeting a 200 bp fragment of the kinetoplast DNA performed at months 0, 9, 15, 18, 21 and 24. When a dog died from leishmaniasis before M21 or M24 (natural death or euthanasia for ethical reasons), the last result available was reported until M24. The number of parasites per ml of bone marrow could thus be calculated, and the results are presented as the geometric mean for each group. Samples were considered as negative if the parasite load was inferior to 40 parasites per ml bone marrow. Error bars represent the SEM. The two groups were significantly differently over the duration of the study (p = 0.035).

Finally, in addition to the differences seen in clinical outcome, the mean bone marrow parasite load over the course of the study was significantly lower in the vaccinated group (p = 0.035) as presented in figure 10.

## Discussion

One of the strengths of this study was the regular detailed assessments performed for each dog. The repeated use of each test further increases confidence in the classification system used. There are no pathognomonic signs in CanL; clinical signs frequently seen in cases of CanL can also be seen in many other diseased conditions [9]. Hence, longitudinal follow-up using several parameters is required for confident interpretation of the status of the dog at the end of the study.

The addition of culture analysis to the longitudinal follow-up further increased our ability to detect the active progressive infections at an earlier stage. Culture is more sensitive than direct visualisation of the parasite in smears, and in this study we also observed that dogs consistently became culture positive in advance of significant rises above the 1/160 threshold in the IFAT titres in the control group. The IFAT titres could not be used for the classification system in the challenge phase due to the fact that vaccinated dogs may develop elevated IFAT titres due to vaccine-induced antibodies (as previously published [32], [33], and as seen in the vaccinal phase of this study). However in all cases the arrival of symptomatic disease was preceded by an obvious sharp increase in the IFAT titres. The sensitivity of the tests used in this study to detect infection were therefore PCR>Culture>IFAT (≥1/160)>Clinical signs.

In this study we observed that the patterns of progression of the infection behaved in a very similar manner to that which has been previously well described [11], [43], and more recently confirmed in a large group of more than 300 naïve dogs [36]. In a highly endemic area, large numbers of dogs may be PCR positive and never progress to the clinical stages of the disease [9]. Dogs that were PCR positive only (subpatent) were able to revert to the *Leishmania*-free status on occasions during this study, and this was more frequently seen in the vaccinated group than the control group. Transient PCR positive states were not unexpected in the vaccinated group, and are consistent with the mode of action of a vaccine. While it is not the objective of the vaccine to prevent the initial infection, it was interesting to note that in the vaccinated group 41% (17/41) of dogs remained negative by PCR on each occasion, in contrast to only 28% (11/39) of control dogs. Although this does not reach statistical significance it could be interesting to follow this parameter in a larger number of dogs to assess if this effect may also be relevant in vaccinated dogs. In any future attempt to study this effect, it would also be important to use “deep” tissues such as bone marrow or lymph nodes, as we did in this study. This would avoid the potential confounding effects of *Leishmania* entry and immediate killing that may be seen when skin samples are examined.

However once a dog became culture positive in this study, it never regressed back to being culture negative, and once a dog developed clinical signs these did not resolve spontaneously, confirming the progressive nature of the disease. This is slightly different to what was seen in an artificial challenge model study previously performed with this vaccine [33]. In that study, some dogs that reached asymptomatic active infection in the vaccinated group did finally manage to resolve this and return to a culture and PCR negative state. The difference probably lies in the fact that in this study there was continued high level natural challenge in contrast to a single time-point intravenous challenge. It is still possible that with large numbers of dogs in the field there could be the possibility of occasional reversion from an actively infected state in vaccinated dogs.

The serological profile of the response to primary vaccination with LiESP/QA-21 was also in line with previously published studies performed with this vaccine [31], [32]. There was a consistent seroconversion, and a predominant IgG2 profile against both ESP and PSA. However, as there was no obvious difference in the antibody profile between resistant and susceptible vaccinated dogs, it is not possible to use such assays to assess the response to vaccination and to predict the success of vaccination in any given dog. The seroconversion detected with IFAT after vaccination and before exposure to infection confirms previously published results [31]–[33] and also confirms that care must be taken in the interpretation of IFAT results in vaccinated dogs.

The sites were chosen specifically to maximise the probability of achieving the elevated *L. infantum* transmission levels required for a strong natural challenge. Indeed by the end of the natural challenge phase, 72% of the control dogs had demonstrated infection, and 90% had demonstrated serological evidence of *Leishmania* exposure, thus confirming the expected high infection pressure in the study sites. In a conventional population around 1/3 of the dogs can be assumed to be susceptible to developing the disease over the course of their lives [9], and in this study we already had 1/3 of the control dogs in a state of active infection within 2 years of the first exposure. It is of course possible that beagles are slightly more susceptible to developing the disease than other breeds, and this is one potential limitation of the study, in common with most other canine vaccine studies. However as the dogs had the same characteristics in both groups this will have no impact on the efficacy data obtained. Furthermore, the randomisation of the dogs by sex and litter will have reduced to the minimum the impact of the dog as a variable. This is important in a study performed in a reasonably restricted number of dogs.

Having a restricted number of dogs is a potential limitation for any study such as this. We saw that several parameters had p-values close to the threshold of significance, and the limited number of dogs is almost certainly a factor in this. However for welfare reasons it is important not to use excessive numbers of dogs, especially when there are repeated sampling procedures and no treatment is given to infected dogs.

The results obtained in this study also confirm the general consensus that even if low IFAT titres (one or two dilution steps below the laboratory threshold) are highly specific (especially in an area where trypanosomes are absent) and can be used as evidence of exposure to the parasite [18], they are not correlated with infection. Several animals had such low titres, but were never demonstrated to be infected by PCR. It is quite probable that these are naturally highly resistant animals that were capable of rapidly killing the parasite before any spread to “deep” tissues such as bone marrow.

The efficacy of the vaccine can be expressed in several ways. It is possible to describe the percentage efficacy and percentage of protection found in a given study. However these results are tied to the particular conditions of the study. In this case the high challenge rates mean that the vaccine was being tested at the upper end of the range of possible challenge levels.

It is also important to note that due to the major differences in the way in which the three vaccines available on the market have been studied, it is not possible to draw any meaningful comparisons about the relative effectiveness of the products available. In the case of the two vaccines available in Brazil, the available efficacy data was obtained under very different conditions, and with study designs that also differed significantly [29], [30]. This is also the case for the prototype vaccine LiESAp/MDP for which a successful efficacy study was published [45]. This inherent complexity in the study of vaccinal prevention of CanL suggests that for the future it could be interesting to have a more standardised accepted protocol for vaccine efficacy investigations. Unfortunately, while use of a standardised protocol allows a reduction in variable factors to permit a more confident interpretation of the results, it also represents a key limitation when applying the results to the general population with variable use of repellent products, living conditions, and breeds.

The use of the odds ratio is an attempt to describe the effects of the vaccine in terms that allow a degree of extrapolation of the effects found in this study to areas with different initial risk levels. In addition, the individual level of risk for any given dog is virtually impossible to determine as even within local areas the level of risk is not homogeneous due to the varying presence of other infected animals and annual variations in climatic factors that impact the vector populations.

While it is clear that vaccination is not able to prevent all cases of the disease, the fact that when the disease developed in vaccinated animals it was generally slower and with milder initial signs is also useful. The majority of deaths due to this disease are the result of progressive, severe, irreversible kidney damage [9]. If the development of the disease can be slowed in cases where it cannot be prevented, this increases the possibility of applying early treatment with better longer term results.

These results have clearly shown that the studied vaccine, when used according to the recommended protocol, conferred protection by reducing the risk of active infections and clinical disease. This is important in terms of the primary objective of vaccination: reducing the incidence of a potentially fatal disease in immunised dogs. However, as the dog's level of infectivity to the vector is also correlated with the development of the later stages of the disease [21], any reduction in the number of dogs suffering from these more advanced, progressive forms of the disease could have a beneficial impact on an epidemiological scale in areas with widespread use of the vaccine. To balance this statement, it must also be noted that despite the very strong correlation between earlier stages of the disease and the lack of ability to infect sand flies, it is not always the case and rarely even subpatent dogs could potentially transmit the parasite. This was seen in a previously published study which nevertheless confirmed a decrease in the infectivity of those LiESP/QA-21 vaccinated dogs that progressed to active infection stages [21]. As the vaccine does not provide complete protection to 100% of dogs in the conditions tested here, it seems wise to combine the use of the vaccine with measures such as anti-feeding repellent/insecticide products. This will increase the ability of the immune system to control any infection received while decreasing the level of challenge that it must control. It is rational to assume that this combined approach is likely to provide the maximum possible level of protection currently available.

While it was essential to minimise the number of variables that could interfere with an accurate assessment of the vaccine efficacy, it will also be important in the future to follow the impact of the vaccine in a large number of dogs of various breeds and ages over a longer period of time. It would also be interesting to follow this work with an assessment of the benefit of a combined vaccination plus insecticide approach, and to assess further the impact of the vaccine when used on a large scale in areas where the zoonotic risk is extremely high.

### Conclusion

The results of this natural challenge trial demonstrate that the LiESP/QA-21 vaccine, when administered according to the recommended protocol (a primary vaccination course of three doses at three-week intervals followed by annual booster vaccinations) provides a significant reduction of the number of actively infected animals and a significant reduction of the probability of developing symptomatic disease. In those animals developing the disease despite vaccination, the progression is generally slower and the disease is generally less severe. The use of a natural challenge study design involving regular in-depth assessments of each dog over time to assess the efficacy is also validated.
