# Correction: A Randomised, Double-Blind, Controlled Efficacy Trial of the LiESP/QA-21 Vaccine in Naïve Dogs Exposed to Two *Leishmania infantum* Transmission Seasons

**DOI:** 10.1371/journal.pntd.0003408

**Published:** 2014-11-20

**Authors:** The PLOS Neglected Tropical Diseases Staff

## Notice of Republication

This article was republished on November 6, 2014, to include the correct versions of Figures 5 and 6. Tables 1 and 2 in the original article were also changed into Figures 4 and 7, respectively, in the republished article. Please download this article again to view the correct version. The originally published, uncorrected article and the republished, corrected article are provided here for reference.

## Supporting Information

File S1 Originally published, uncorrected article.
(PDF)

File S2 Republished corrected article.
(PDF)
